# Ecological impact of the end-Cretaceous extinction on lamniform sharks

**DOI:** 10.1371/journal.pone.0178294

**Published:** 2017-06-07

**Authors:** Rachel A. Belben, Charlie J. Underwood, Zerina Johanson, Richard J. Twitchett

**Affiliations:** 1 Department of Earth Sciences, Natural History Museum, London, United Kingdom; 2 Department of Earth and Planetary Sciences, Birkbeck, University of London, London, United Kingdom; University of Michigan, UNITED STATES

## Abstract

Lamniform sharks are apex marine predators undergoing dramatic local and regional decline worldwide, with consequences for marine ecosystems that are difficult to predict. Through their long history, lamniform sharks have faced widespread extinction, and understanding those ‘natural experiments’ may help constrain predictions, placing the current crisis in evolutionary context. Here we show, using novel morphometric analyses of fossil shark teeth, that the end-Cretaceous extinction of many sharks had major ecological consequences. Post-extinction ecosystems supported lower diversity and disparity of lamniforms, and were dominated by significantly smaller sharks with slimmer, smoother and less robust teeth. Tooth shape is intimately associated with ecology, feeding and prey type, and by integrating data from extant sharks we show that latest Cretaceous sharks occupied similar niches to modern lamniforms, implying similar ecosystem structure and function. By comparison, species in the depauperate post-extinction community occupied niches most similar to those of juvenile sand tigers (*Carcharias taurus*). Our data show that quantitative tooth morphometrics can distinguish lamniform sharks due to dietary differences, providing critical insights into ecological consequences of past extinction episodes.

## Introduction

The end-Cretaceous mass extinction event witnessed the loss of 40% of marine genera and is ranked as the fifth most severe such event of the Phanerozoic [1]. Globally, 43% of elasmobranch genera went extinct within the Maastrichtian [2]. Among neoselachian sharks, 34% of genera and 45% of species became extinct [3]. Marked regional differences exist in the magnitude of loss, with widespread extinction in Tethys but relatively little in the Pacific [4].

Simply documenting rates of taxonomic extinction does not, however, provide a measure of ecological impact, the magnitude of which is decoupled from the associated loss of taxa during past events [1,5]. Understanding ecological impact on ecosystem structure and function is critical in predicting future change [6,7]. In terms of its ecological impact, the end-Cretaceous event was the second most severe crisis of the Phanerozoic [1]. Studies of predatory bony fish demonstrate significant ecological selectivity during the end-Cretaceous extinction [8,9], but no similar study has been attempted for sharks even though they represent an ideal model group for such analyses. Due to their hardness, mineral and histological composition, and polyphyodont dentitions [10], shark teeth are very common in the rock record. Furthermore, individual tooth morphology reflects prey type, feeding strategy and ecological role, for example as top predators within the community [11,12].

Our study used novel morphometric analyses of assemblages of fossil teeth spanning the Cretaceous/Palaeogene (K/Pg) boundary in Morocco to quantify the ecological impact of the end-Cretaceous mass extinction event on Lamniformes (mackerel sharks). Our analyses demonstrate that Maastrichtian lamniforms were ecologically diverse, occupying similar ecomorphospace compared to extant taxa, whereas post-extinction Danian lamniforms were significantly smaller with much reduced ecological diversity.

## Materials and methods

### Fossil materials

Disarticulated fossil shark teeth were collected at several sites in Morocco (S1 Fig). The Danian assemblage was collected from oolitic phosphorites at Gara Tilda in western Morocco (31° 33′ 57″ N, 008° 45′ 4″ W), about 3.5–4 metres above the base of the Danian, part of the Meskala Basin; and at private farmland north-east of Khouribga in northern Morocco (32° 55′ 20″ N, 006° 46′ 31″ W), about 1–1.5 metres above the base of the Danian, part of the Ouled Abdoun Basin. The Maastrichtian assemblage was collected from Bakrit in the Middle Atlas (33° 3′ 39″ N, 005° 8′ 52″ W), dry and wet sieved from a weathered phosphate peloid-rich black mudstone. Whilst fossiliferous Maastrichtian rocks are present at all sites, only at Bakrit was the lithology suitable for bulk sampling in order to collect large and unbiased samples of lamniform teeth. The Meskala and Ouled Abdoun basins in Morocco are part of the Mediterranean-Tethyan Phosphogenic Belt, which spans around 50 million years of discontinuous occurrences, from the Turonian to the Eocene in Morocco, the phosphate deposits start in the Maastrichtian and end in the late Ypresian [13], and were created in shallow and warm epicontinental seas [14].

The palaeobathymetry of these basins has not been studied in detail, but all sites are likely to represent moderately deep water with direct connection to the open Tethyan Ocean. Phosphatic mudstones at Batrit contain a very similar lamniform fauna to those of similar age from elsewhere in northern Morocco. Additional horizons higher in the Maastrichtian succession probably represent very shallow water deposition, but these were not sampled in this study. The oolitic phosphorites of sites other than Bakrit yield a diverse assemblage containing frequent specimens of taxa which are usually associated with deeper water, such as the Hexanchidae. Thus, all of the sampled horizons and sites are considered to represent similar pelagic water depths and palaeoenvironments.

There is no evidence for significant biostratinomic sorting at any site, with well-preserved vertebrate remains ranging from sub-millimetre shark and ray teeth to large tetrapod bones. In addition, articulated specimens of larger vertebrates are present at all levels. Species assignments largely followed Arambourg [15], taking into account more recent taxonomic changes (eg Noubhani and Cappetta, 1997 [16]). All fossil specimens are housed in the Natural History Museum, London.

### Ethics statement

No permits were required for the described study, which complied with all relevant regulations.

### Modern materials

Modern teeth were sourced from the collections of the Natural History Museum, London, and Birkbeck University, London, and consist of the complete, articulated jaws from nine individuals: adults of *Carcharias taurus* (sandtiger shark), *Carcharodon carcharias* (white shark), *Isurus oxyrinchus* (shortfin mako), *Odontaspis ferox* (small tooth sand tiger), *Alopias pelagicus* (pelagic thresher), and *Pseudocarcharias kamoharai* (crocodile shark), and new-born juveniles of *C*. *taurus*, *I*. *oxyrinchu*s and *C*. *carcharias*. These were chosen to represent the entire range of morphologies present in modern, non-planktivorous, lamniform taxa that live in shelf seas analogous to the depositional setting of the fossil sites.

### Methods

**Fig 1:**
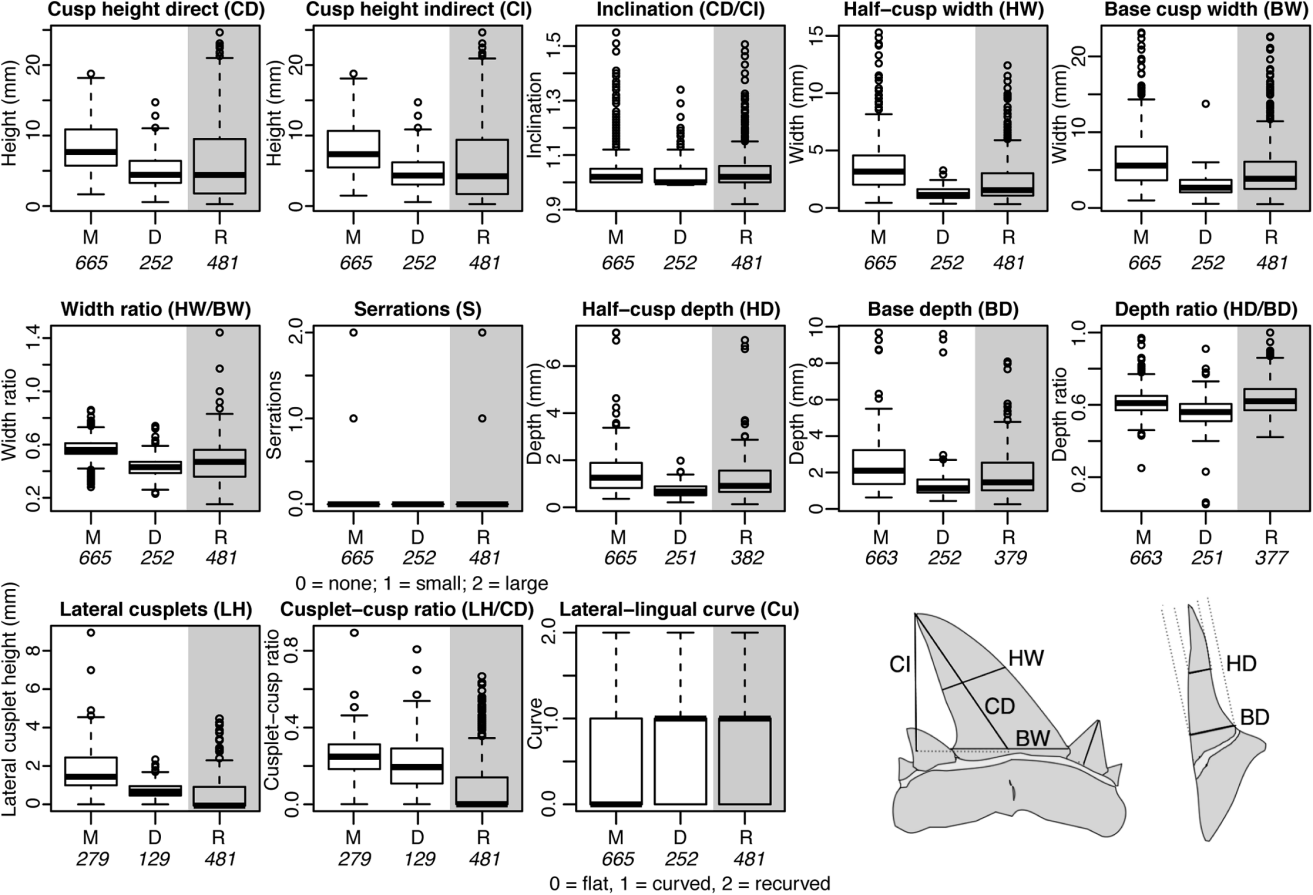
Comparative morphometrics of Maastrichtian, Danian and Recent lamniform teeth. Thirteen variables were analysed: seven characters were measured directly (A-G), from which five other variables were calculated, and the presence of small (1) or large (2) serrations were recorded. CD = direct cusp height, CI = indirect cusp height, HW = half-cusp width, BW = base cusp width, BD = base depth, HD = depth at half-cusp height, LH = height of largest lateral cusplet. M = Maastrichtian, D = Danian, R = Recent. Fossil teeth were disarticulated, Recent teeth were measured in situ within jaws (differentiated by grey shading). Italics indicate the number of teeth with complete, measureable characters in each subset.

To quantify tooth shape, thirteen key ecomorphological characters were assessed for each fossil and modern tooth (Fig 1). Nine parameters were measured in order to best describe the morphology with regards to feeding type (e.g. primary prey and feeding behaviour): direct cusp height (CD), indirect cusp height (CI), width at cusp base (BW), width at half-cusp height (HW), depth at cusp base (BD), depth at half-cusp height (HD), height of the largest lateral cusplet (LH), presence and type of serrations (S), and curvature of the labial face of the tooth (Cu). From these, four additional variables were calculated: inclination (I) from CD/CI, width ratio (WR) from HW/WB, depth ratio (DR) from DHC/DBC, and cusp-cusplet ratio (CC) from LH/CD. S and Cu are categorical. For S, 0 = no serrations, 1 = fine/microscopic serrations, 2 = large serrations. For Cu, 0 = no curve, 1 = curved lingually, 2 = tip curved labially. Definition of lateral cusplets and serrations followed that of Bemis *et al*. [17]. The same measurements were made on fossil and modern teeth.

For larger fossil teeth (greater than ~2.5mm direct cusp height) and all modern teeth, measurements were made using digital callipers accurate to 0.01mm. For smaller fossil teeth (less than ~2.5mm direct cusp height) measurements were taken using Axio Zoom and Olympus BX61/63 microscopes. All fossil lamniform shark teeth with complete cusps were measured and analysed in this study (n = 917; S1 Table).

For the modern teeth, in each case the complete functional series was measured, i.e. the labial-most teeth at each position in the jaw. If the teeth of the functional series, and the first and second replacement series, had incomplete cusps or lacked their lateral cusplets, that position in the jaw was skipped. In order to prevent damage to the specimens, some parameters, such as base depth, could not be measured on some teeth that were too close together in the jaw. In total, 481 modern teeth were measured (S2 Table).

Statistics and figures were all produced using the software R [18]. The ordination method Principal Component Analysis (PCA), using a singular value decomposition of the data matrix, was used for visualisation of patterns in the multivariate data. It also allowed the ranking of individual variables in their relative importance in the overall morphology. Only those fossil and modern teeth that preserved all measured parameters were subjected to PCA. Multivariate Analysis of Variance (MANOVA) was used to test the differences between the Danian, Maastrichtian, and modern samples, while a modified ANOVA for unbalanced group sizes, non-normality and heteroscedasticity [19] was used to test individual variables between the assemblages.

## Results

### Pre-extinction, Maastrichtian assemblage

**Fig 2:**
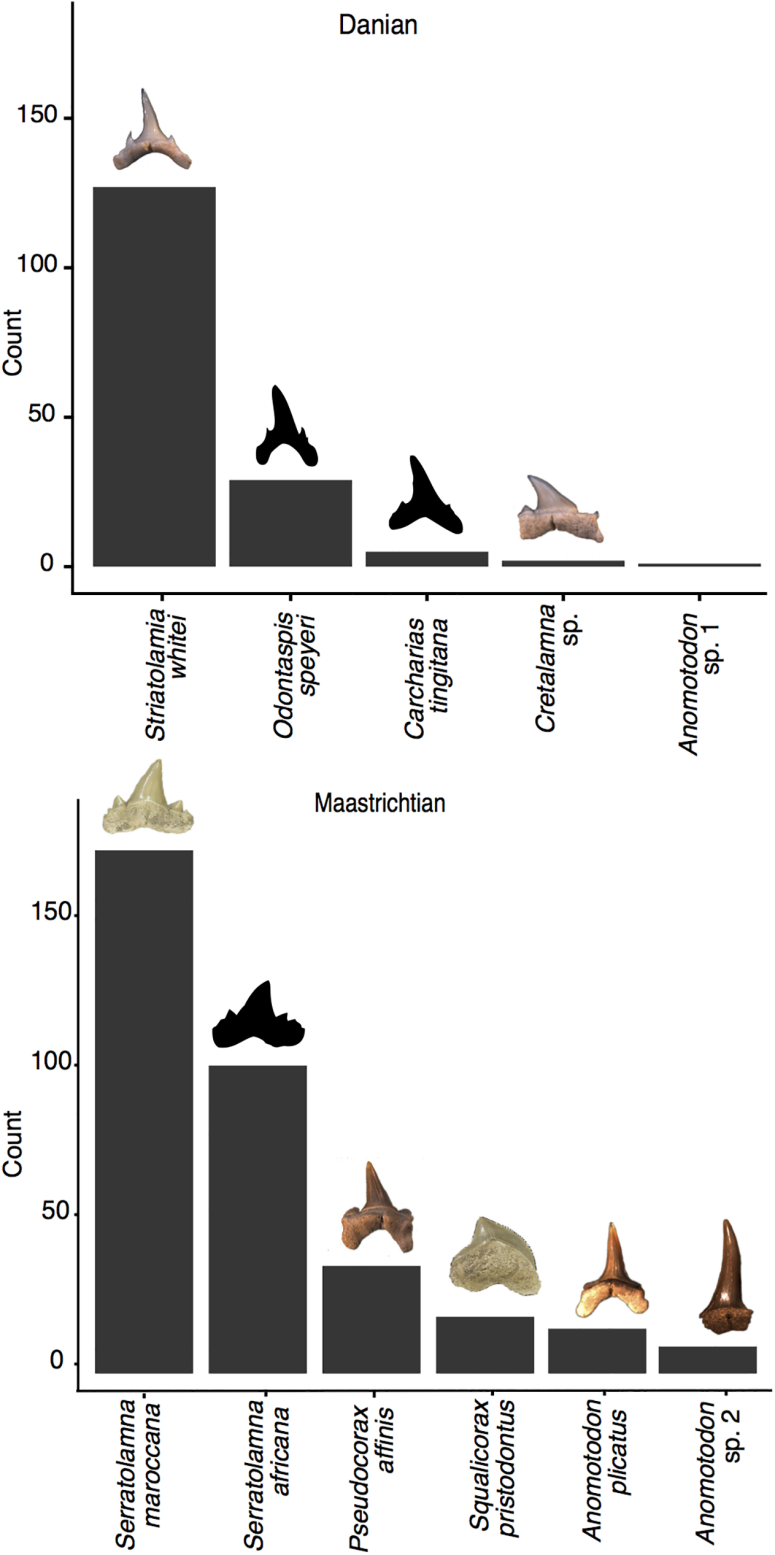
Relative abundances of Maastrichtian and Danian fossil lamniforms. Numbers comprise only those teeth that are complete and identifiable to species.

In total, the Maastrichtian assemblage comprises 665 teeth, of which 358 (54%) are identifiable to species, representing 6 species from 4 genera and 4 families (Fig 2 and S3 Table). Only one of the genera (*Anomotodon*) is also recorded in the Danian assemblage. Whilst another genus (*Cretalamna*) is known to occur in Maastrichtian and Danian facies [20], it is very rare in the former and not encountered in this study. The disappearance of the more common Maastrichtian taxa cannot be attributed to preservation or sampling biases because they are represented by large, robust teeth (e.g. *Serratolamna maroccana* and *Squalicorax pristodontus*), unlikely to be preferentially lost during diagenesis or overlooked during collection. *Pseudocorax affinis*, *Squalicorax prisotodontus* and *Serratolamna africana*, have no post-Cretaceous records, becoming extinct at the K/Pg boundary. Records of *Anomotodon plicatus*, *Serratolamna maroccana* and *S*. *serrata* in younger strata [3], are probably a result of reworking [20] and not survival. Of the lamniform families recorded in the Maastrichtian in this study, only the Anacoracidae became extinct at the end of the Cretaceous [3,11].

### Post-extinction, Danian assemblage

The Danian assemblage consists of 252 teeth, of which 164 (65%) are identifiable, representing 5 species from 5 genera and 3 families (Fig 2 and S3 Table). All Danian species appear to have originated after the K/Pg boundary [15,20]; there is no overlap of species between the Maastrichtian and Danian in this study. The status of species of *Cretalamna* remains uncertain but the Danian species appears different from Cretaceous specimens. The other extant family sampled in this study, Lamnidae, also originated after the K/Pg boundary [3].

### Morphometric analysis

**Fig 3:**
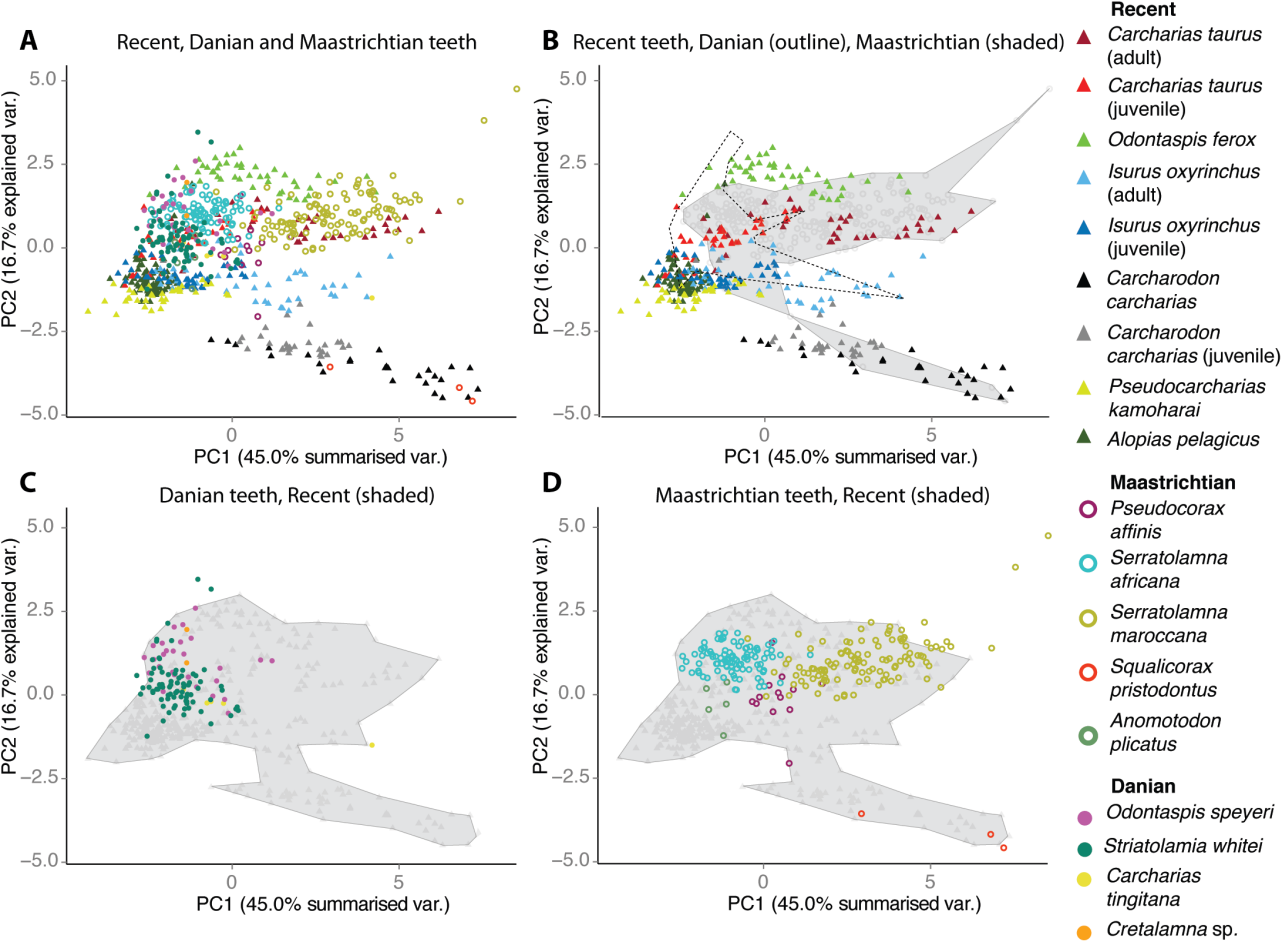
Tooth morphospace of Maastrichtian, Danian and Recent lamniform sharks. (a) Principal Components Analysis (PCA) of all complete, identifiable teeth; (b) morphospace occupied by teeth of individual Recent lamniforms compared to that occupied by the Maastrichtian assemblage (grey shading) and Danian assemblage (dotted outline); morphospace occupied by teeth of Danian (c) and Maastrichtian (d) lamniforms compared to that occupied by Recent lamniforms (grey shading).

Teeth of Danian and Maastrichtian lamniform sharks are morphologically distinct and significantly different from each other, and from Recent species (Fig 1; S4–S6 Tables). Only two of the thirteen morphometric variables (i.e. labial-lingual curve and tooth inclination) record no change through the K/Pg boundary. Danian lamniform teeth lack serrations and are significantly smaller and narrower than those of the Maastrichtian (Fig 1). The Maastrichtian assemblage has greater disparity and occupies a more extensive morphospace along the two principal component axes that summarise the most variation (Fig 3). Principal Component 1 corresponds to tooth size and robustness, indicating that the Danian teeth are no larger than the smaller Maastrichtian teeth. PC2 corresponds to lateral cusplet height, labial-lingual curve, width ratio, and the presence and type of serrations, indicating that the Danian teeth had smaller lateral cusplets, and had no serrations (S7 Table). Danian teeth are also significantly smaller than those of modern lamniforms, and differ from modern teeth in every measured variable except inclination and labial-lingual curve (Fig 1; S4 and S5 Tables). Even within a single family (i.e. the ‘Odontaspididae’), significant differences are recorded between the Danian and Recent in all variables except inclination and labial-lingual curve (S4 and S5 Tables).

## Discussion

Absence of larger-toothed sharks in the post-extinction assemblage mirrors global changes recorded in teleosts, where large-bodied taxa with fast-snapping jaws containing large, fang-like teeth also disappeared at the K/Pg boundary [9]. Body size reduction during past extinction events has been recorded in a range of marine organisms, reflecting a temporary dwarfing of surviving taxa (i.e. the Lilliput effect), and/or the temporary disappearance or preferential extinction of large-bodied taxa [21]. A shift to smaller body size is a predicted response of marine animals to global warming [22] with top predators potentially suffering most as they are also exposed to indirect effects of reduced prey size [23]. In addition to bony fish, a wide range of marine invertebrates, including molluscs [24] and infaunal crustaceans [25], as well as planktic foraminifera [26] are smaller in the aftermath of the end-Cretaceous extinction than in pre-extinction communities. This suggests that body size reduction affected every trophic level, with implications for ecosystem function [22,27]. Body size reduction in fish has been recorded in the aftermaths of other past extinction events too, such as after the end-Devonian event [28].

Lamniformes, common top predators in Danian marine ecosystems, were not only significantly smaller than those in pre-extinction ecosystems, but also lacked the diversity of tooth morphologies recorded in the Maastrichtian. Given that the morphology of shark teeth is closely associated with their role in feeding [11,12], though not necessarily in a clear-cut manner [29,30], our results demonstrate that the end-Cretaceous extinction event led to significant ecological shifts as well as major taxonomic turnover amongst lamniforms. Reduction in morphological disparity of teeth may indicate changes in available prey, and/or in the ecology of the predators, which may have wider implications for ecosystem function. Our morphometric analyses of the teeth of extant lamniforms of known ecology provide a means of addressing this and a quantitative, comparative method of determining the feeding habits of the fossil taxa.

Significant differences in all tooth morphometric characters are recorded between the nine individual Recent sharks and between the six extant species (S8 and S9 Tables). Ordination plots reveal that teeth of individual sharks cluster in morphospace, with little overlap between most taxa (Fig 3). This is consistent with either an ecological or phylogenetic control on tooth-shape. Critically, however, there are intraspecific differences, with the teeth of adults and juveniles of the same species occupying different morphospace. This is not just due to obvious differences in size, but probably reflects known ontogenetic shifts in dietary preference of the species *C*. *taurus*, *C*. *carcharias* and *I*. *oxyrinchus* analysed in this study [31–33], supporting assumptions that ecology is the main control on tooth shape in living Lamniformes.

Teeth of Maastrichtian lamniform species occupy a very similar morphospace to those of modern species (Fig 3). Given that this clustering is not due to close phylogenetic relationships, because there are different genera in the two datasets, we conclude that it is the result of similar ecology, feeding habit and prey choice, and hence the ecology of the living species may be used to interpret the fossils. Thus we infer that *S*. *pristodontus* was a Maastrichtian apex predator equivalent of the white shark, *C*. *carcharias*, with a diet of large vertebrate prey [33]. Large individuals of *C*. *carcharias* derive a considerable proportion of their diet from scavenging whale carcasses [33], and similarly *Squalicorax* commonly scavenged marine reptile carcasses [34]. The extinction of almost all marine reptiles [35] and large teleost fish [9] at the K/Pg boundary may have led directly to the extinction of *S*. *pristodontus*. In Morocco the Maastrichtian phosphorites are especially rich in remains of large marine reptiles and teleosts, and the disappearance of both is especially evident [15,36].

The teeth of *Serratolamna maroccana* overlap in morphospace with those of adult *C*. *taurus* (Fig 3), implying that it too was an opportunist that consumed a wide range of demersal prey, mostly teleosts and elasmobranchs, up to half its own body length in size, with some crustaceans and squid [31]. Most Danian teeth occupy a very similar morphospace to teeth of juvenile *C*. *taurus*. One major shift during ontogeny is that adult *C*. *taurus* consume more benthic elasmobranchs such as batoids [37]. We infer that Danian lamniform sharks probably also consumed a wide range of small-sized, demersal prey as noted above, but probably not batoids and other benthic elasmobranchs.

The morphospace occupied by the Danian assemblage does not overlap with that of adult *C*. *carcharias*, indicating continued absence of top apex predators feeding on large pelagic vertebrates. Likewise, almost no overlap between the Danian assemblage and the teeth of *O*. *ferox*, *A*. *pelagicus* and *P*. *kamoharai* suggests that lamniform sharks with similar feeding habits were not part of the local, post-Cretaceous community. *Odontaspis ferox*, an outer shelf lamniform, has smaller and less robust teeth than *C*. *taurus* (inner shelf), with relatively large lateral cusplets, and a diet composed of demersal teleosts, squid and shrimp that are smaller, slower moving and softer-bodied than the prey of *C*. *taurus* [38]. *Alopias pelagicus* is primarily an open ocean species and consumes mainly small teleosts and squid [39]. The diet of the deeper water *P*. *kamoharai* is poorly known, but comprises mainly small teleosts, crustaceans and coleoids [38]. The near absence of Danian teeth with similar morphologies to those of *O*. *ferox*, *A*. *pelagicus* and *P*. *kamoharai* may reflect local rarity of more pelagic/outer-shelf teleosts, crustaceans and coleoids after the end-Cretaceous extinction event.

## Conclusions

Quantitative morphometric analyses of modern lamniform shark teeth using the key variables measured in this study are able to discriminate between individuals of different species and ontogenetic stage due to differences in their feeding habits and ecology. When applied to fossil assemblages this becomes a powerful tool for inferring the ecology of extinct species as well as providing insights into the potential availability of prey, even when those prey are soft-bodied and not preserved in the rock record. This approach provides a more rigorous means of inferring the ecology of extinct taxa than qualitative methods, and can be used to study the ecological impact of past extinction and environmental change on marine ecosystems. We predict that this approach will be applicable to other shark taxa and other past events, and could be applied to historical collections to provide empirical data of the state of marine ecosystems prior to human disturbance.

## Supporting information

S1 Fig Sedimentary logs and sample horizons of Maastrichtian-Danian rocks exposed at the three sample sites. Sections logged and specimens collected by CJU.
(TIF)

S1 Table Fossil lamniform morphometric data. CD = direct cusp height; CI = indirect cusp height; I = inclination; HW = width at half-cusp height; BW = width at cusp base; WR = width ratio; S = presence and type of serrations; BD = depth at cusp base; HD = depth at half-cusp height; DR = depth ratio; Cu = curvature of the labial face of the tooth; LH = height of the largest lateral cusplet; CC = cusp-cusplet ratio. For S, 0 = no serrations, 1 = fine/microscopic serrations, 2 = large serrations. For Cu, 0 = no curve, 1 = curved lingually, 2 = tip curved labially. All measures are in mm.
(XLSX)

S2 Table Recent lamniform morphometric data. CD = direct cusp height; CI = indirect cusp height; I = inclination; HW = width at half-cusp height; BW = width at cusp base; WR = width ratio; S = presence and type of serrations; BD = depth at cusp base; HD = depth at half-cusp height; DR = depth ratio; Cu = curvature of the labial face of the tooth; LH = height of the largest lateral cusplet; CC = cusp-cusplet ratio. For S, 0 = no serrations, 1 = fine/microscopic serrations, 2 = large serrations. For Cu, 0 = no curve, 1 = curved lingually, 2 = tip curved labially. All measures are in mm.
(XLSX)

S3 Table Numbers of taxa identified in the fossil assemblages, and those used as Recent comparisons. Revised from Arambourg (1952), with Arambourg originals as following: ^1^Genus: *Lamna*, ^2^Genus: *Odontaspis*.
(XLSX)

S4 Table Results of statistics associated with Fig 1. Results for mean, standard deviation, standard error, and confidence intervals associated with Fig 1.
(XLSX)

S5 Table Results of Danian, Maastrichtian and Recent Multiple Comparisons of Means: Tukey Contrasts, testing individual variables. Bold text is used to highlight non-significant variables. For Odontaspididae (Danian and Recent), all species of Odontaspididae from the Danian were tested against all species of Odontaspididae of the Recent individuals.
(XLSX)

S6 Table Results of MANOVA, testing pairwise differences between whole assemblages. Pillai is Pillai’s trace, a test statistic where the larger the value, the greater the contribution to the model. Bold text is used to highlight non-significant values. For Odontaspididae (Danian and Recent), all species within Odontaspididae from the Danian were tested against all species within Odontaspididae from the Recent individuals.
(XLSX)

S7 Table Characters that influence the first three Principle Component (PC) axes. Values show the cumulative proportion of the first three PC axes and PC loadings: the closer to 0, the less influence the variable has on the PC. Bold text is used to highlight values contributing the most to each PC.
(XLSX)

S8 Table Results of Recent specimen ANOVA, testing individual variables. Individuals shows comparisons between all nine Recent jaws, and Taxa shows comparisons when pooling adult and juvenile of the same species. Bold text is used to highlight non-significant values.
(XLSX)

S9 Table Results of Recent specimen MANOVA, testing pairwise differences. Individuals shows comparisons between all nine Recent jaws, and Species shows comparisons when pooling adult and juvenile of the same species. Pillai is Pillai’s trace, a test statistic where the larger the value, the greater the contribution to the model.
(XLSX)
