# Supplementary figures and images for: Ecological impact of the end-Cretaceous extinction on lamniform sharks
Source: PLoS One. 2017 Jun 7;12(6):e0178294. doi: 10.1371/journal.pone.0178294 (PMC5462355; doi:10.1371/journal.pone.0178294)

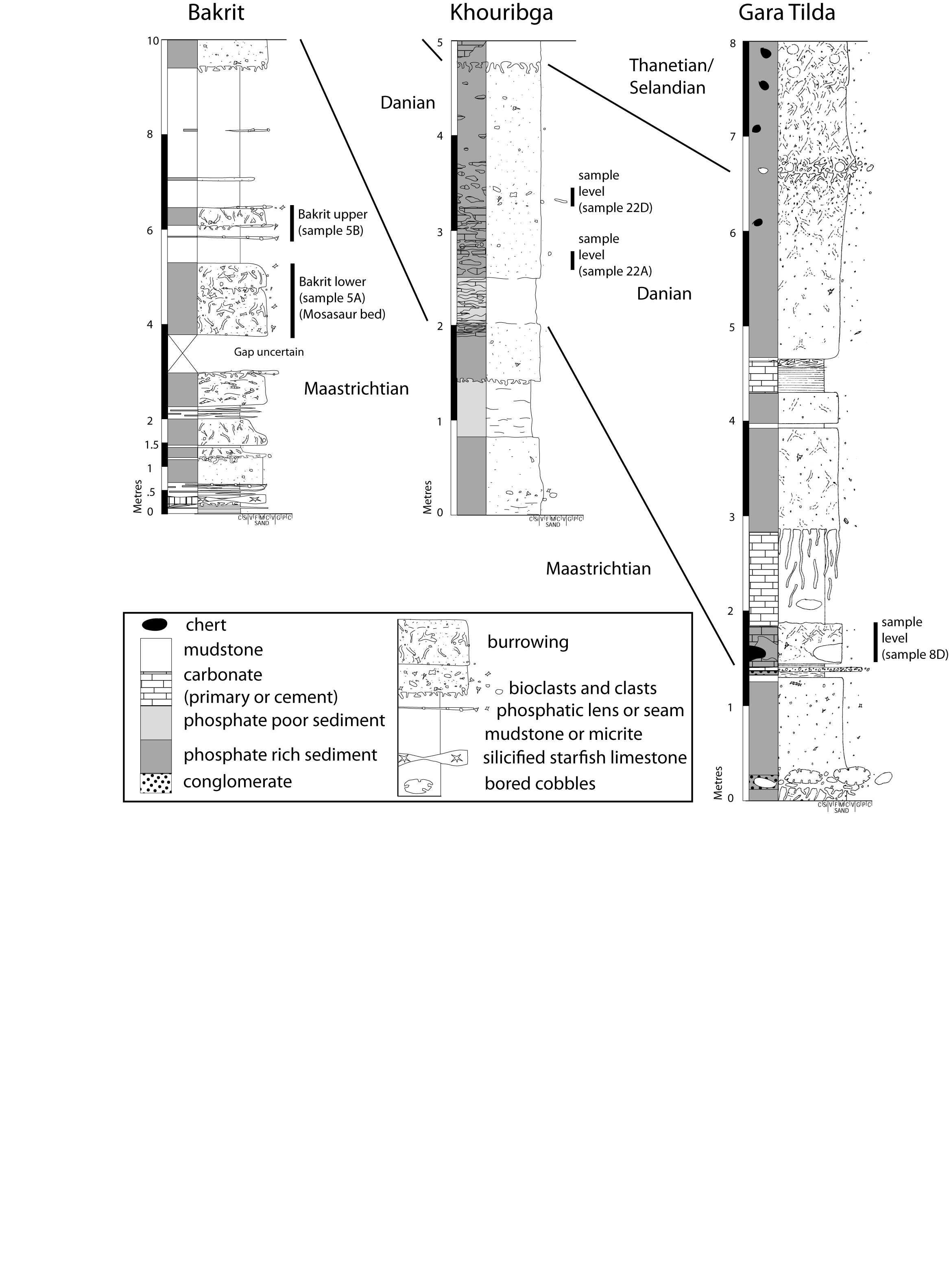

Supplement: S1 Fig — Sections logged and specimens collected by CJU. (TIF) [file pone.0178294.s001.tif]
